# Air Breathing Cathodes for Microbial Fuel Cell using Mn-, Fe-, Co- and Ni-containing Platinum Group Metal-free Catalysts

**DOI:** 10.1016/j.electacta.2017.02.033

**Published:** 2017-03-20

**Authors:** Mounika Kodali, Carlo Santoro, Alexey Serov, Sadia Kabir, Kateryna Artyushkova, Ivana Matanovic, Plamen Atanassov

**Affiliations:** a Center Micro-Engineered Materials (CMEM), Department of Chemical and Biological Engineering, University of New Mexico, Albuquerque, NM, USA; b Theoretical Division, Los Alamos National Laboratory, Los Alamos, NM 87545, USA

**Keywords:** Microbial Fuel Cells, Oxygen Reduction Reaction, PGM-free catalysts, Fe-AAPyr, High Power Generation

## Abstract

The oxygen reduction reaction (ORR) is one of the major factors that is limiting the overall performance output of microbial fuel cells (MFC). In this study, Platinum Group Metal-free (PGM-free) ORR catalysts based on Fe, Co, Ni, Mn and the same precursor (Aminoantipyrine, AAPyr) were synthesized using identical sacrificial support method (SSM). The catalysts were investigated for their electrochemical performance, and then integrated into an air-breathing cathode to be tested in “clean” environment and in a working microbial fuel cell (MFC). Their performances were also compared to activated carbon (AC) based cathode under similar conditions. Results showed that the addition of Mn, Fe, Co and Ni to AAPyr increased the performances compared to AC. Fe-AAPyr showed the highest open circuit potential (OCP) that was 0.307 ± 0.001 V (vs. Ag/AgCl) and the highest electrocatalytic activity at pH 7.5. On the contrary, AC had an OCP of 0.203 ± 0.002 V (vs. Ag/AgCl) and had the lowest electrochemical activity. In MFC, Fe-AAPyr also had the highest output of 251 ± 2.3 μWcm^−2^, followed by Co-AAPyr with 196 ± 1.5 μWcm^−2^, Ni-AAPyr with 171 ± 3.6 μWcm^−2^, Mn-AAPyr with 160 ± 2.8 μWcm^−2^ and AC 129 ± 4.2 μWcm^−2^. The best performing catalyst (Fe-AAPyr) was then tested in MFC with increasing solution conductivity from 12.4 mScm^−1^ to 63.1 mScm^−1^. A maximum power density of 482 ± 5 μWcm^−2^ was obtained with increasing solution conductivity, which is one of the highest values reported in the field.

## Highlights

• PGM-free catalysts were synthesized using sacrificial support method.
• Catalysts were made with Fe, Co, Mn and Ni as metal center and AAPyr as precursor.
• Fe-catalysts showed highest performance for ORR in microbial fuel cell.
• Increase in solution conductivity led to a maximum power of 482 ± 5 μWcm^−2^

## Graphical abstract

**Figure:**
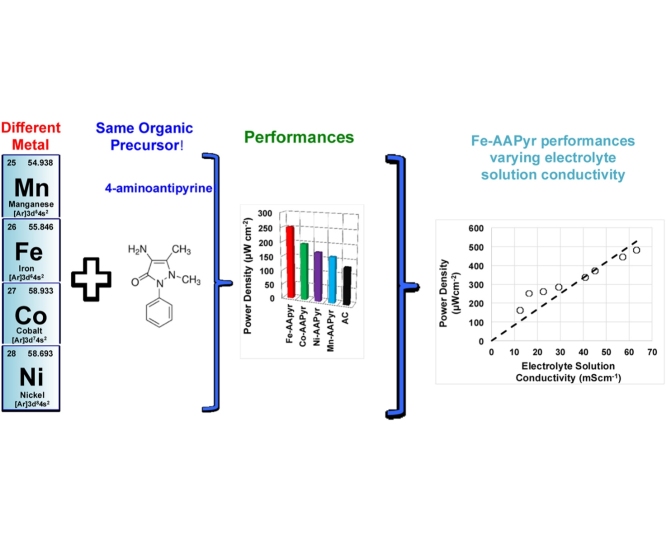

## 1 Introduction

Microbial fuel cells (MFCs) are bio-electrochemical systems that can treat wastewater while simultaneously generating electricity. This co-generative configuration can theoretically replace the existing energy-intensive treatments plants [1], [2]. Unfortunately, the performances of MFCs are limited by serveral factors that hinder its large-scale application [3], [4], [5]. It has been established that one of the main factors limiting the power output of MFCs is the reduction reaction in the cathode [6]. The most used oxidant at the cathode is oxygen, and this is due to the fact that oxygen has a high reduction potential, and is naturally abundant in the atmosphere. Several issues concerning the oxygen reduction reaction (ORR) are as follows: i) high overpotentials; ii) low kinetics and iii) high ohmic resistances of the existing cathodes [6]. It has been shown that enzymes have low activation overpotentials within 100 mV, [7], [8], [9] but their utilization in a polluted environment is prohibitive due to their rapid degradation and deactivation [10]. From the theoretical open circuit potential (OCP) which − at pH 7 − is 606 mV vs. Ag/AgCl (3 M KCl), the activation overpotentials reported using metal based catalysts are roughly 300 mV [11] that can be further increased up to 400 mV when activated carbon (metal-free) is used [12] and even to 500–600 mV when different carbonaceous or steel materials are used [13], [14]. The activation overpotentials enormously contribute to initial losses concerning the electroreduction of oxygen in neutral media.

While ORR pathways have been widely studied in both acidic [15], [16], [17], [18] and alkaline [19], [20], [21], [22] media, the kinetic mechanisms taking place in neutral media − in which MFCs usually operate − are not fully understood. Using the rotating ring disk electrode (RRDE) technique, it was recently shown that oxygen is reduced via a 2e^−^ mechanism on carbon black and activated carbon [23], [24], where as a 4e^−^ pathway is dominant for Pt [24] and Fe-based catalyst [25], [26]. In order to enhance the ORR kinetics, usually three different pathways can be selected for integration of catalysts into the cathode layer. The first one is the utilization of high surface area carbons like activated carbon (AC) [27], [28], graphene-based materials [29], carbon nanotubes (CNTs) [30], carbon nanofibers (CNFs) [31] or nitrogen doped carbon [32]. Activated carbon seems to be the best compromise between cost and performances and lately it has been largely utilized as a cathode in MFCs [32]. The second approach is based on usage of Pt or Pt-based materials named as platinum group metals (PGMs) as cathode catalysts. These catalysts were extensively used in the past, but both the high costs and low durability have significantly lowered their utilization in MFCs [33]. Particularly, Pt is poisoned severely with anions (mainly S^2−^ and SO_4_^2−^) which are naturally presented in the wastewater as recently demonstrated [34].

The third and more emerging category is the utilization of PGM-free catalysts [33], [35], [36] based on M-N-C materials in which M is a transition metal, N is nitrogen and C is carbon. PGM-free catalysts have been heavily studied, and several catalysts containing Fe [37], [38], [39], [40], [41], Mn [42], [43], [44], Co [45], [46], [47] and Ni [48], [49] have been used in MFCs. Despite slightly higher cost in cathode manufacturing, the utilization of PGM-free catalysts guarantees higher performances compare to AC, and it assures higher durability in long-terms operations [50], [51]. In previous studies, we showed that sprayed Fe-Aminoantipyrine (Fe-AAPyr) cathode outperformed AC and Pt during both linear scan voltammetry in clean media and in a working MFC [50]. More recently, we showed that high performances were achieved by Fe-N-C cathode synthesized from different organic precursors (namely, Ricobendazole and Niclosamide), and high stability output was demonstrated during 32 days of durability test [51].

In the current literature, to the best of our knowledge, there are no a clear and direct comparisons that have been made among the PGM-free ORR catalysts fabricated with different transition metals (Mn, Fe, Co and Ni). Comparison of existing literature on PGM-free catalysts in MFCs is quite complicated, and this is because the catalysts are often synthesized using different fabrication methods and precursors and the performances are only compared to AC or Pt. Moreover, diverse working conditions lead to further differences in the output and the comparison becomes even harder to determine. Last but not least, it has not been well established which transition metal (M) among the M-N-C PGM-free catalysts − Fe, Co, Mn and Ni − has the superior electrochemical performances towards the electroreduction of oxygen in neutral media.

In view of that, we have −for the first time- made a direct comparison of Fe-, Co-, Mn- and Ni- based PGM-free cathode catalysts that were synthesized using the same method and organic precursor (Aminoantipyrine, AAPyr) under the same working conditions. Particularly, in this study, we studied the electrochemical performance of the above mentioned catalysts using linear sweep voltammetry (LSV) in neutral media, where AC was used as the benchmark. Then the performance of these catalysts incorporated into an air-breathing cathode was evaluated in running MFCs with exactly the same operating conditions. Once the best performing catalyst was identified, solution conductivity of the working MFC was increased, and effect of solution conductivity on power curves and anode/cathode polarization curves was carried out to determine the one of the highest performing M-N-C catalysts in MFCs

## 2 Experimental

### 2.1 Catalysts preparation

The PGM-free catalysts were made using the sacrificial support method (SSM) previously described [52], [53]. Particularly, the metal’s salt (Fe(NO_3_)_3_·9H_2_O, Co(NO_3_)_2_·6H_2_O, Mn(NO_3_)_2_·4H_2_O, Ni(NO_3_)_2_·6H_2_O) was separately wet impregnated with aminoantipyrine (AAPyr) on the surface of fumed silica (Cab-O-Sil M5; surface area: ∼ 250 m^2^ g^−1^). AAPyr − a rich source of carbon and nitrogen – was used as an organic precursor. The mixture was then ultrasonicated and dried overnight at roughly 85 °C. The obtained materials were then ground to fine powder using ball milling. The powder was then subjected to heat treatment under Ultra High Purity nitrogen (flow rate 100 mL min^−1^) atmosphere. The temperature was increased from room temperature to 950 °C with a rate of 25 °C min^−1^. Once the desired temperature was reached, pyrolysis took place for 30 minutes. The silica was then etched and removed from the obtained pyrolyzed M-N-C materials using hydrofluoric acid (20 wt%). The catalysts were then extensively washed with de-ionized water and dried overnight at 85 °C. The catalysts obtained were named as a function of the metal used: Mn-AAPyr, Fe-AAPyr, Co-AAPyr, Ni-AAPyr.

### 2.2 Catalysts Surface Chemistry

The synthesized materials − Mn-AAPyr, Fe-AAPyr, Co-AAPyr, Ni-AAPyr were characterized catalysts using X-ray fluorescence (XRF). XRF was conducted to identify the metals in the catalysts using an EDAX Orbis with a Rh tube source and a titanium adsorption edge filter. Samples were measured in a vacuum with a filament voltage of 15 kV and a current of 200 μA. Data are reported on the Supporting Information.

High-resolution XPS was performed using Kratos Ultra DLD spectrometer. Al Kα monochromatic source at 225 W was utilized. No charge compensation was necessary. High-resolution C 1s, O 1s, N 1 s and corresponding metal spectra (Fe2p/Co2p/Ni2p/Mn2p) were acquired from three areas per sample at a pass energy of 20 eV. CASAxps was used to process spectra. The Linear background was used for quantifying C 1s, O 1 s and N1 s spectra and Shirley background for metal spectra. Three areas per sample were analyzed and the average is presented. The morphologies of the synthesized materials were determined by scanning electron microscopy (SEM, Hitachi S-5200) with an accelerating voltage of 20 keV).

### 2.3 Electrochemical measurements

The electrochemical activity of the synthesized Mn-AAPyr, Fe-AAPyr, Co-AAPyr, Ni-AAPyr catalysts as well as activated carbon (AC) was investigated using a three-electrode cell configuration comprising of a catalyst coated rotating disc electrode (glassy carbon disk) as the working electrode, a graphite rod as the counter electrode and Ag/AgCl (3 M KCl) as the reference electrode. The working electrodes were prepared by depositing sonicated inks formulated using a mixture of a binder (150 μL of 0.5 wt% Nafion solution), 850 μL of water: isopropanol mixture (4:1 volumetric ratio)) and 5 mg of the catalyst of interest. Inks were then deposited on to the 0.2475 cm^2^ glassy carbon disk through drop casting technique with a catalyst loading of 200 μg cm^−2^. The disk was then immerged into the oxygen saturated electrolyte with a pH of 7.5 prepared using 0.1 M potassium phosphate (K-PB) and 0.1 M KCl. Linear sweep voltammograms were then obtained between +0.5 V (vs.Ag/AgCl) to −0.7 V (vs. Ag/AgCl) with at a scan rate of 5 mV s^−1^ and the disc rotation speed set at 1600 RPM.

### 2.4 Cathode preparation

The cathodes were prepared by pressing a mixture activated carbon (AC, Norit SX Ultra, Sigma Aldrich), PTFE (60 wt% solution, Sigma Aldrich) and Carbon black (CB, Alfa Aesar) in the ratio of 70:20:10 respectively with M-N-C catalysts. CB was added in order to enhance the conductivity of the formed pellet [54]. 40 ± 1 mgcm^−2^ of above mentioned base materials (AC:CB:PTFE) and 2 ± 0.1 mgcm^−2^ of catalysts (Fe-AAPyr, Ni-AAPyr, Mn-AAPyr, Co-AAPyr) were subjected to mechanical press with a pressure of 2.5mT (metric tons) for span of 5 minutes on a stainless steel mesh (SS, McMaster), which acted as a current collector [51]. Cathodes with a geometric area of 2.85 cm^2^ were exposed to the solution by mounting on the later hole of the glass tube MFC.

### 2.5 Cathode polarization curve in “clean” media

A modified Pyrex glass bottle with an empty volume of 125 mL was used as an electrochemical cell. A lateral hole was built in order to accommodate the cathode that was screwed on it leaving the current collector (SS) exposed to the atmosphere and the catalytic layer exposed to the liquid electrolyte. In order to attain stable electrode potential, remove adsorbed oxygen and increase materials wettability for enhancing the three-phase interface (TPI), the cathodes were exposed to the potassium phosphate buffer (K-PB) solution (0.1 M) with 0.1 M KCl overnight (at least 15 hours). The electrocatalytic activity of these cathodes in clean conditions was done using linear sweep voltammetry (LSV) technique. Three electrode setup was used in which cathode acts as the working electrode, platinum mesh as the counter electrode and Ag/AgCl 3 M KCl (+210 mV vs. SHE) as a reference electrode. LSVs were run in a range between open circuit potential and −0.4 V vs. Ag/AgCl (3 M KCl) at a scan rate of 0.2 mVs^−1^.

### 2.6 Microbial fuel cell construction

After the electrochemical measurement, the cathode was inserted in a working microbial fuel cell and left in open circuit voltage (OCV) for at least 3 hours till the output was stabilized. The MFC was a similarly modified Pyrex glass bottle (volume of 125 mL) single chamber with a lateral hole to accommodate the cathode. The cathode was in air breathing configuration with the metallic mesh exposed to air and the catalytic side facing directly the solution with no membrane separator utilized. Passive air was used. Carbon brush (3 cm as diameter and 3 cm as height, Millirose, USA) was used as the anode of the MFC. The anodes used were already fully working from running experiments [55]. The MFC solution was based on a mixture of 50:50 ratio K-PB (0.1 M and 0.1 M KCl) and activated sludge (Albuquerque Southeast Water Reclamation Facility, New Mexico, USA), along with 3 g L^−1^ of sodium acetate (NaOAc) as an organic substrate. All the measurements were done at room temperature (22 ± 1 °C) in Albuquerque (NM, USA) at roughly 1600 AMSL. It is well known that at this altitude, atmospheric pressure is 20% less than at sea level, and this certainly affects the presence negatively and partial pressure of oxygen, therefore, the ORR is adversely penalized.

### 2.7 Microbial Fuel Cell Electrochemical Measurements

After the cathode was inserted in a working MFC and left to stabilize for few hours, overall polarization curves were run from open circuit voltage to 0 mV with a scan rate of 0.2 mV s^−1^. Particularly, the anode was used as a counter electrode, the cathode as working electrode and the reference channel was short-circuited with the counter. Simultaneously, anode potential and cathode potential were recorded on another potentiostat (Biologic SP-50, France) channel. Power was calculated by multiplying current and voltage (P = V x I) from the polarization curve. Power density and the current density was calculated with respect to the cathode geometric area (2.85 cm^2^). Every electrochemical experiment was run in triplicates.

### 2.8 Long term stability tests

Durability tests were conducted over a period of one month in which voltage over an external resistance of 100 ohm was recorded using a data log system (Personal DAQ/56). Duplicate and separate MFCs were run. The solution used was the same as described before with 50% in volume of 0.1 M K-PB and 0.1 M KCl and 50% in volume of activated sludge. In order to have longer cycles, 4 g L^−1^ was added in each cycle. At day 15, the solution was completely refreshed. Power curves were run also at the end of the experiments to estimate the degradation of the MFCs in terms of performances.

### 2.9 Solution conductivity analysis

Once the catalyst with the highest performance was identified, the solution conductivity of the working MFC was varied between 12.4 mS cm^−1^ and 63.1 mS cm^−1^. Particularly, K-PB was prepared using K_2_HPO_4_ and KH_2_PO_4_ in concentration of 0.05 M, 0.1 M, 0.2 M, 0.3 M, 0.5 M, 0.6 M, 0.8 M and 1 M. The pH was 7.5 and it was adjusted using KCl or KOH. Each different K-PB concentration also contained 0.1 M KCl, and it was blended in 50% volume with activated sludge (AS). The solution conductivity had the values are reported in Table 1. Also, in this case, polarization curves were run from open circuit voltage to 0 mV (0.2 mV s^−1^ scan rate) with the anode as a counter electrode, the cathode as working electrode (reference short-circuited with the counter). Anode potential and cathode potential during the polarization were recorded separately.

## 3 Results and Discussion

### 3.1 Surface Chemistry and Morphology of the catalysts investigated

**Fig. 1:**
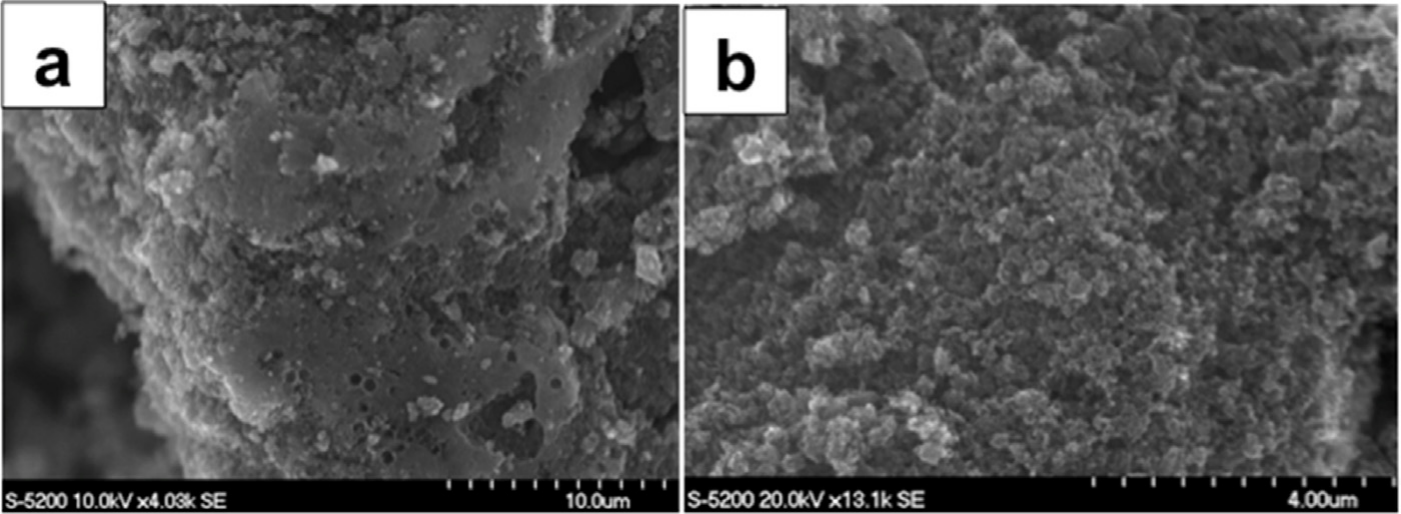
Scanning electron micrographs of Fe-AAPyr synthesized using SSM.

Scanning Electron Microscopy was used to analyze the morphology of the synthesized materials. Fig. 1 a-b shows the SEM micrographs of the Fe-AAPyr catalyst synthesized using the sacrificial support method (SSM). As it can be seen from this figure, the porous morphology of the Fe-AApyr catalyst is clearly visible- as it is with all catalysts that have been previously fabricated using the sacrificial support method (SSM) [50], [51], [52], [53]. These pores were formed during the etching of the amorphous fumed silica template which was fused into the metal precursor-carbon-nitrogen matrix during wet impregnation (see catalyst preparation, section 2.1). EDS analysis of these materials also indicated that the active sites containing metal were atomically dispersed in the catalyst matrix, as the % of the dispersed metal actives was <1 wt% in all the samples (data not shown). Only Fe-AAPyr is here presented but also Ni-AAPyr, Co-AAPyr and Mn-AAPyr had very similar morphology.

X-Ray Fluorescence was used to determine the presence of the anticipated transition metal in the M-N-C catalysts being investigated. It can be noticed (Fig. S1) that in each catalyst, the only metal used during the synthesis was identified by the specific peak in the graph for each catalyst. No other metals were identified in the graphs indicating the absence of metallic contamination during the synthesis process. XRF technique is very selective and was used in the present study as an only qualitative method in order to determine the presence of metals within ppb concentration.

**Fig. 2:**
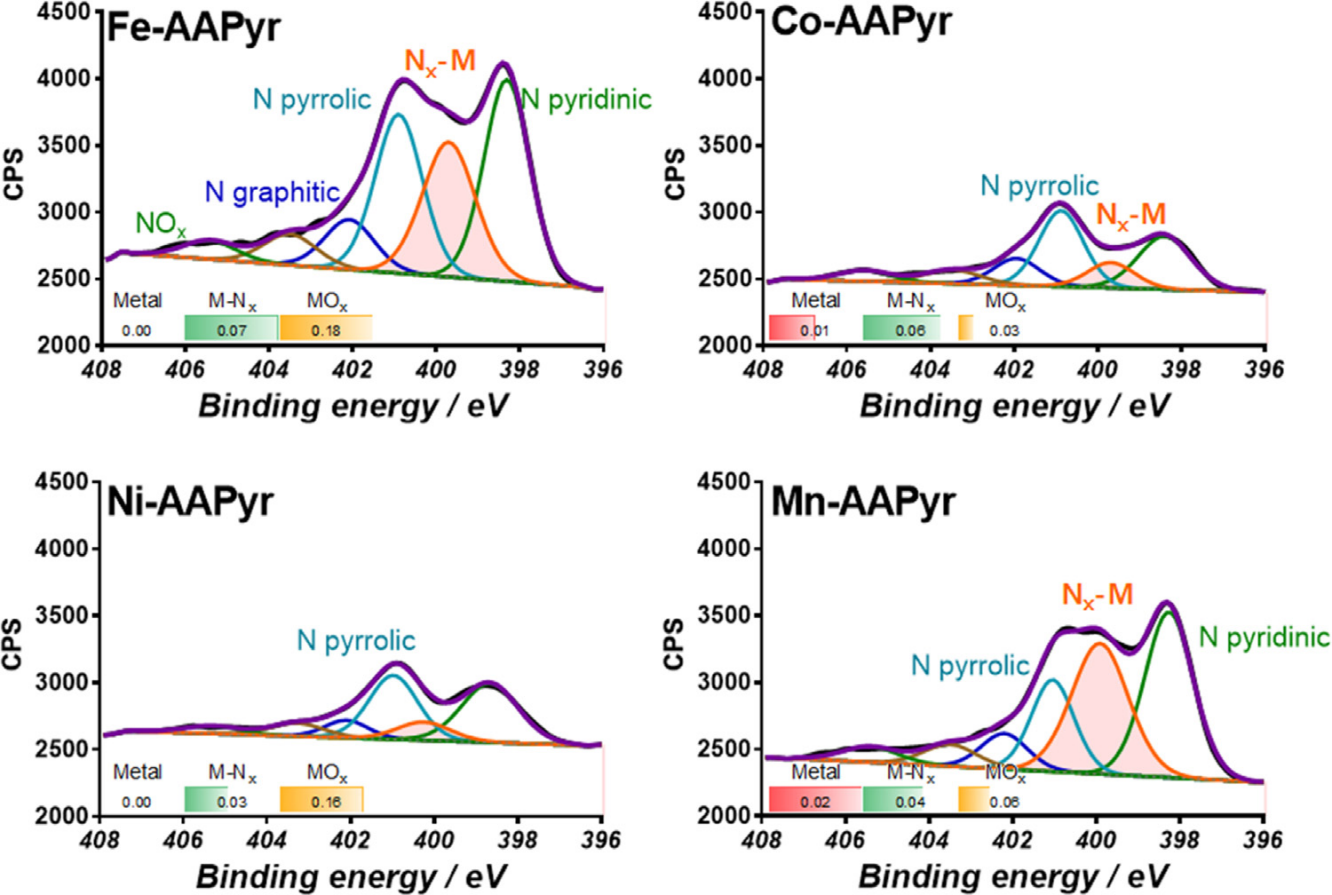
High-resolution N1 s spectra for 4 catalysts. Atomic percentage of different types of metals obtained from Fe 2p, Co 2o, Ni 2p and Mn 2p high resolution spectra is shown for each material.

The chemical composition of 4 electrocatalysts was studied in detail by XPS. Table 2 shows elemental composition and chemical speciation for carbon, nitrogen, and metal. Fig. 2 shows high-resolution N 1 s spectra for materials with four different metals for comparison along with atomic percentage of each type of metal present obtained from corresponding high resolution spectra of metal, i.e. Fe 2p, Co 2p, Ni 2p and Mn 2p (Fig. S2). It was shown previously that N moieties have a positive effect on oxygen reduction reaction [56], [57].

Overall chemical composition varies a lot from sample to sample. It is important to mention that the same loadings of nitrogen precursor and metal precursor were utilized in synthesis of electrocatalysts. The resulted elemental and chemical composition detected is different due to the way metal and nitrogen get incorporated within the carbon matrix during pyrolysis and leaching. Fe-AAPyr has the highest amount of N and metal. Mn-AAPyr has the largest amount of oxygen and nitrogen. The smaller relative amount of metal is detected for this sample, however. Both Co-AAPyr and Ni-AAPyr samples have very similar elemental composition. Carbon chemistry can be separated into three major constituents − graphitic and aliphatic network, carbon-nitrogen defects and surface oxides C_x_O_y_. Co-AAPyr has the highest amount of graphitic carbon and the smallest amount of surface oxides due to a smaller amount of defects in the graphene-like matrix. Smallest amount of C-N defects is correlated with the small amount of nitrogen detected for this sample.

High-resolution spectra have been fitted with 6 peaks [58]. Pyridinic nitrogen at 398.3 eV is largest for Fe- and Mn-AAPyr samples. A peak that corresponds to nitrogen coordinated with metal has the highest intensity for the same two samples. The fitting of this peak was done based on the density functional theory calculated binding energies of N 1 s in Fe-N_4_, Co-N_4_, Ni-N_4_, and Mn-N_4_ centers, which are shown in Fig. S3, by following the procedure in [59], [60]. N 1 s binding energies were calculated as 399.7 eV, 399.6, 399.9, and 400.0 eV for Fe-N_4_, Co-N_4_, Ni-N_4_, and Mn-N_4_ centers, respectively. Although the fitting of the Metal-N peak was done using the same Metal-N_4_ coordination as an representative (Fig. S3), please note that different coordinations of metal are expected in the material [60], [61]. As it was previously shown on the example of iron and cobalt [60], sites with different coordination of metal are expected to result in relatively narrow distribution of N 1 s binding energies contributing to the same XPS region between pyridinic nitrogen peak at 398.3 eV and pyrrolic nitrogen peak at 401.2 eV.

Pyrrolic nitrogen at 401.2 eV and graphitic N at 402.1 eV are largest for Co- and Ni-AAPyr. Two peaks at highest binding energy are due to NO_x_ species, and they are also largest for Co- and Ni-AAPyr samples. Pyridinic nitrogen and metal coordinated to nitrogen have been reported to be important chemical sites for electrocatalytic reactions [58]. Surface oxides have been shown to be an important measure of defects in carbon network which are also very important for electrocatalytic activity [62]. From this standpoint, Fe-AAPyr and Mn-AAPyr supposed to have very similar highest activity among four samples. The chemical state of metal has been evaluated from relevant high-resolution metallic spectra, Fe 2p, Co 2p, Ni 2p and Mn 2p. Fe-AAPyr and Ni-AAPyr are free from metallic particles which is critical observation (Fig. S2). High amounts of metal oxides are detected for Co- and Fe-AAPyr samples. Both Co- and Mn-AAPyr have a significant amount of metallic particles detected which may be detrimental for electrocatalytic activity.

The best performing sample has a unique balance of chemistry as reported by XPS. Fe-AApyr has the highest amount of total nitrogen, total metal, high amount of pyridinic and nitrogen coordinated to the metal, high amount of defects in carbon and absence of metallic particles. The next performing sample, Co-AAPyr, has very much smaller amount of nitrogen and N-pyridinic and Nx-me, a higher amount of graphitic carbon with fewer defect sites and metallic Co detected. Ni-AAPyr is similar in structural chemistry to Co-AAPyr with the exception of the absence of metallic particles. The smaller absolute amount of Ni-Nx in comparison to Co-Nx results in slightly worse activity than that of Co-AAPyr. Finally, the worst performing sample Mn-AAPyr has very similar type of nitrogen-carbon network that should have resulted in performance close to Fe-AAPyr. The presence of metal Mn particles, however, may be the reason for worse performance.

### 3.2 Electroreduction of Oxygen in Neutral Media

**Fig. 3:**
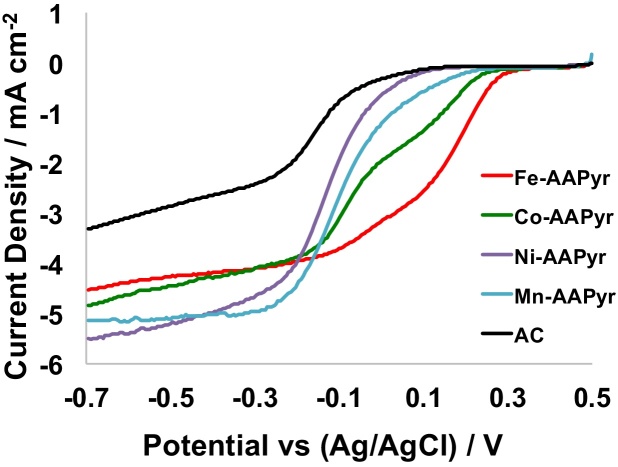
Rotating Disk Electrode Current measured for Mn-AAPyr, Fe-AAPyr, Co-AAPyr, Ni-AAPyr and AC.

Fig. 3 shows the linear sweep voltammograms obtained using a Rotating Disc Electrode (RDE) at 1600 RPM and 5 mV s^−1^ for the synthesized Mn-AAPyr, Fe-AAPyr, Co-AAPyr, Ni-AAPyr catalysts as well as AC in the pH = 7.5 electrolyte saturated with O_2_ at room temperature. The current densities have been normalized to the geometric area of the electrode and potentials in the manuscript are referred to the Ag/AgCl (3 M KCl) electrode. It can be seen that among all synthesized transmission metal catalysts, Fe-AApyr has the highest onset potential of 0.31 V vs. Ag/AgCl. The half wave potentials of the transition metal catalysts also positively shifts from Ni-AApyr (-0.12 V vs. Ag/AgCl) to Fe-AApyr (+0.16 V vs. Ag/AgCl) according to the following trend: Ni-AApyr < Mn-AApyr < Co-AApyr <Fe-AApyr, summarized in Table 3. In contrast, activated carbon not only has the lowest onset and half wave potentials in comparison, but it also has the lowest limiting current densities, indicating that the presence of transition metal active centers are important for the efficient electroreduction of oxygen in neutral media. Among all the synthesized catalysts, Fe-AApyr has the highest electrochemical performance, which also corroborates the polarization curves obtained using MFCs operating with Fe-AApyr as a cathode catalyst.

### 3.3 Cathode Polarization curves

**Fig. 4:**
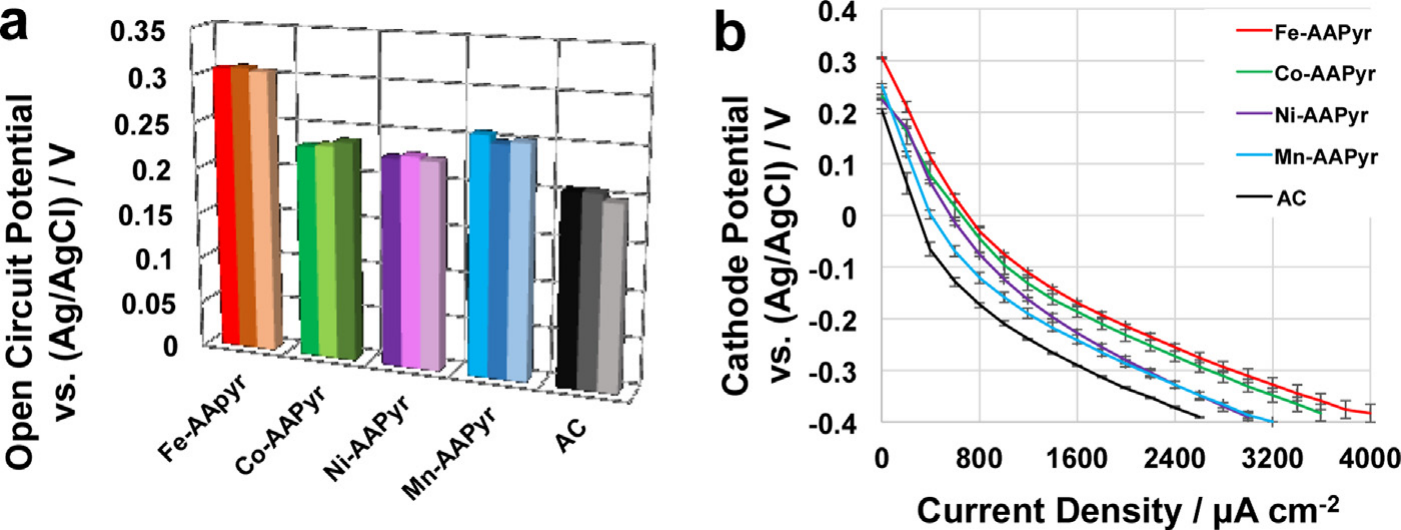
Cathode potentials measured before LSVs (a) and LSV in potassium phosphate buffer (0.1 M) (b).

Three separate linear sweep voltammetries of the air breathing cathodes containing M-AAPyr catalysts were run in K-PB (0.1 M) and 0.1 M KCl (Fig. 4). This investigation was done in order to study the electrocatalytic activity of Fe-AAPyr, Co-AAPyr, Mn-AAPyr and Ni-AAPyr catalysts in neutral media and “clean” conditions. After leaving the cathode exposed to the electrolyte for over 15 hours, it can be determined the open circuit potential of the different cathodes (Fig. 4.a). Particularly, Fe-AAPyr had the highest open circuit potential (0.307 ± 0.001 V vs Ag/AgCl) followed by Mn-AAPyr (0.252 ± 0.004 V vs Ag/AgCl), Co-AAPyr (0.233 ± 0.004 V vs Ag/AgCl), Ni-AAPyr (0.226 ± 0.002 V vs Ag/AgCl) while AC had the lowest open circuit potential that was 0.203 ± 0.004 V (vs Ag/AgCl). These results demonstrated that the utilization of PGM-free catalysts lowers the activation losses compared to AC. Unfortunately, the activation overpotential were still high in the range of 0.28-0.36 V compare to the theoretical value. Fe-AAPyr had the highest electrocatalytic activity followed by Co-AAPyr, Ni-AAPyr, and Mn-AAPyr. AC had the lowest electrocatalytic activity among the material investigated (Fig. 4.b). The current density of 1400 μAcm^−2^ was reached at a potential of −0.142 ± 0.004 V for Fe-AAPyr, −0.162 ± 0.011 for Co-AAPyr, at −0.196 ± 0.005 V for Ni-AAPyr, at −0.216 ± 0.009 V for Mn-AAPyr and at a potential of −0.268 ± 0.002 V for AC. From those results, it can be determined that Fe-AAPyr had the highest open circuit voltage at null current and the highest potential at a measured current. Standard deviation detected was low indicating reproducibility in manufacturing the materials. These results follows the RDE trend (Fig. 3) except Ni-AAPyr and Mn-AAPyr in which the performances are versed when integrated into the cathode.

### 3.4 Performances in MFC

**Fig. 5:**
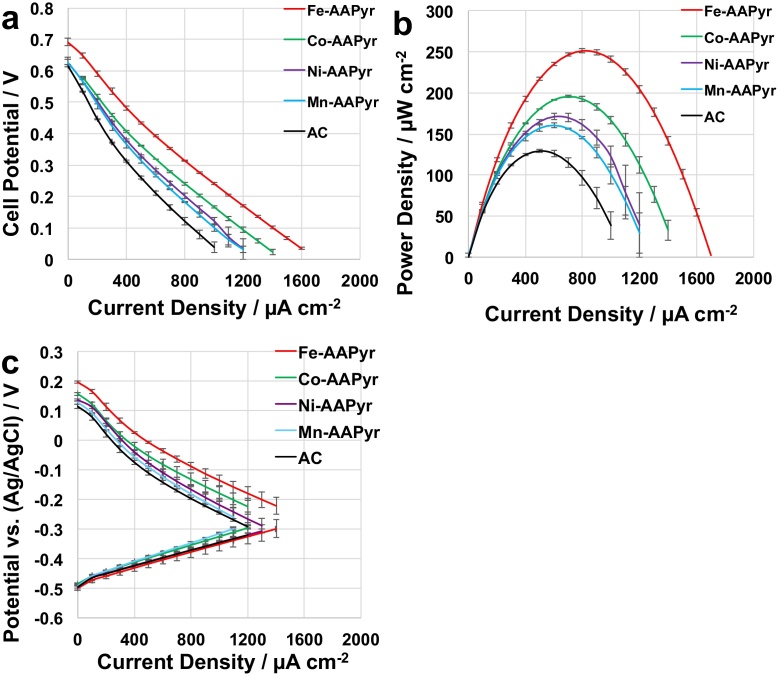
Overall polarization curves (a), power curves (b) and anode and cathode polarization curves of MFC with Fe-AAPyr (red), Co-AAPyr (green), Ni-AAPyr (violet), Mn-AAPyr (light blue) and AC (black) as cathode catalyst.

The cathodes have then been incorporated into running MFCs and polarization curves (Fig. 5.a), power curves (Fig. 5.b), and electrode polarizations have been determined (Fig. 5.c). Polarization curves (V–I) showed different trends that followed the results previously presented for the cathode linear sweep voltammetries (Fig. 4.b). The MFC polarization curves showed highest open circuit voltage and overall performances for Fe-AAPyr. Fe-AAPyr had open circuit voltage of 0.691 ± 0.011 V followed by Co-AAPyr (0.637 ± 0.002 V), Mn-AAPyr (0.628 ± 0.008 V) and by Ni-AAPyr (0.627 ± 0.015 V) (Fig. 4.a). MFC with AC cathode had the lowest open circuit voltage that was 0.616 ± 0.003 V (Fig. 5.a). Power curves showed that Fe-AAPyr had the highest power output that was 251 ± 2.3 μW cm^−2^. The other PGM-free had a maximum power density of 196 ± 1.5 μWcm^−2^ (Co-AAPyr), 171 ± 3.6 μW cm^−2^ (Ni-AAPyr), 161 ± 2.8 μW cm^−2^ (Mn-AAPyr) and 130 ± 4.2 μW cm^−2^ (AC) that was actually the lowest power achieved (Fig. 5.b). Fe-AAPyr had a power density that was 28% higher compared to Co-AAPyr, 48% better than Ni-AAPyr, 54% better than Mn-AAPyr and 102% better compared to AC. Single electrode polarizations taken during the overall polarization curves showed that the difference in performances was caused exclusively by the cathode behavior (Fig. 5.c). The anode polarizations performed similarly as expected (Fig. 5.c). Also, in this case, low standard deviation indicated good reproducibility.

### 3.5 Durability Tests in Microbial Fuel Cells

**Fig. 6:**
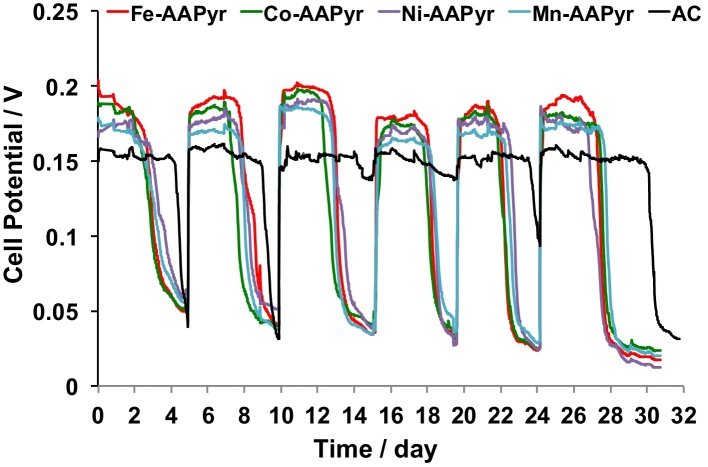
Voltage recorded during the durability tests.

Duplicate MFCs were run for over one month and the voltage was recorded continuously. The average values of the recorded voltage are here presented (Fig. 6) while the duplicate voltage for MFC having different cathode catalyst is presented in the Supporting Information (Fig. S4). It can be noticed that generally Fe-AAPyr outperformed the other cathode catalysts along the entire experimentation (Fig. 6). Similarly, after Fe-AAPyr, Co-AAPyr had higher voltage compared to Ni-AAPyr and Mn-AAPyr. The latter was the worst performing metal-based catalyst during the durability test. All the M-AAPyr had higher performances compared to AC cathodes MFCs (Fig. 6). Interestingly, the cycles of M-AAPyr cathode catalysts MFCs were shorter than the cycles of AC cathode MFC probably indicating a faster consumption of fuel due to higher voltage/current generated (Fig. 6).

**Fig. 7:**
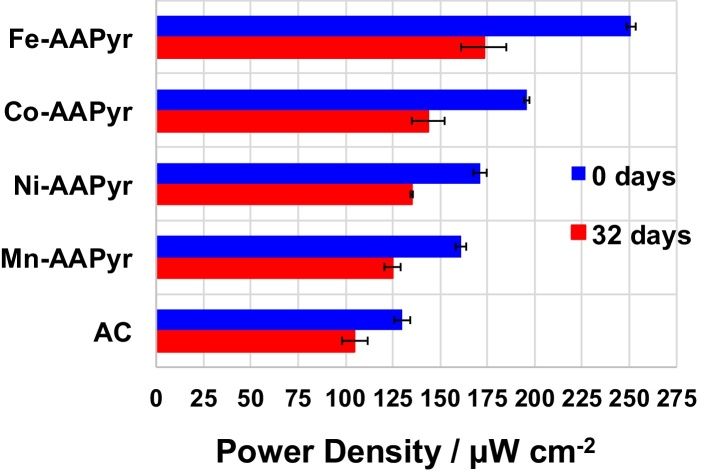
Variation in power peaks during the 32 days investigation.

Power curves were also measured at the end of the durability test and presented into the Supporting Information (Fig. S5). The differences in power peaks between the beginning and the end of the tests are here reported (Fig. 7). Generally, the power peak decreases over time due to the biological growth on the cathode [63], the precipitation of carbonates that enhance the proton mass transfer resistance [64] and the negative effect of the interaction between the catalyst and the pollutants contained into wastewater [50], [51]. In our case, clear biofilm/fouling was formed on the cathode surface as showed by Fig. S6. The power peak decreased by 30% in the case of Fe-AAPyr (from 251 ± 2.3 μW cm^−2^ to 175 ± 12 μW cm^−2^), 26% for Co-AAPyr (from 196 ± 1.5 μW cm^−2^ to 144 ± 8.7 μW cm^−2^), 21% for Ni-AAPyr (from 171 ± 3.6 μW cm^−2^ to 135 ± 0.6 μW cm^−2^), 22% for Mn-AAPyr (from 161 ± 2.8 μW cm^−2^ to 125 ± 4.1 μW cm^−2^) and 19% for AC (from 130 ± 4.2 μW cm^−2^ to 107 ± 6.7 μW cm^−2^). Those data are in agreement with previously reported data in which the decrease in M-N-C catalyst varied between 10 and 30% during onemonth investigation [50], [51], [63].

### 3.6 Performances of Fe-AAPyr catalysts in working MFCs with electrolyte having different solution conductivity

**Fig. 8:**
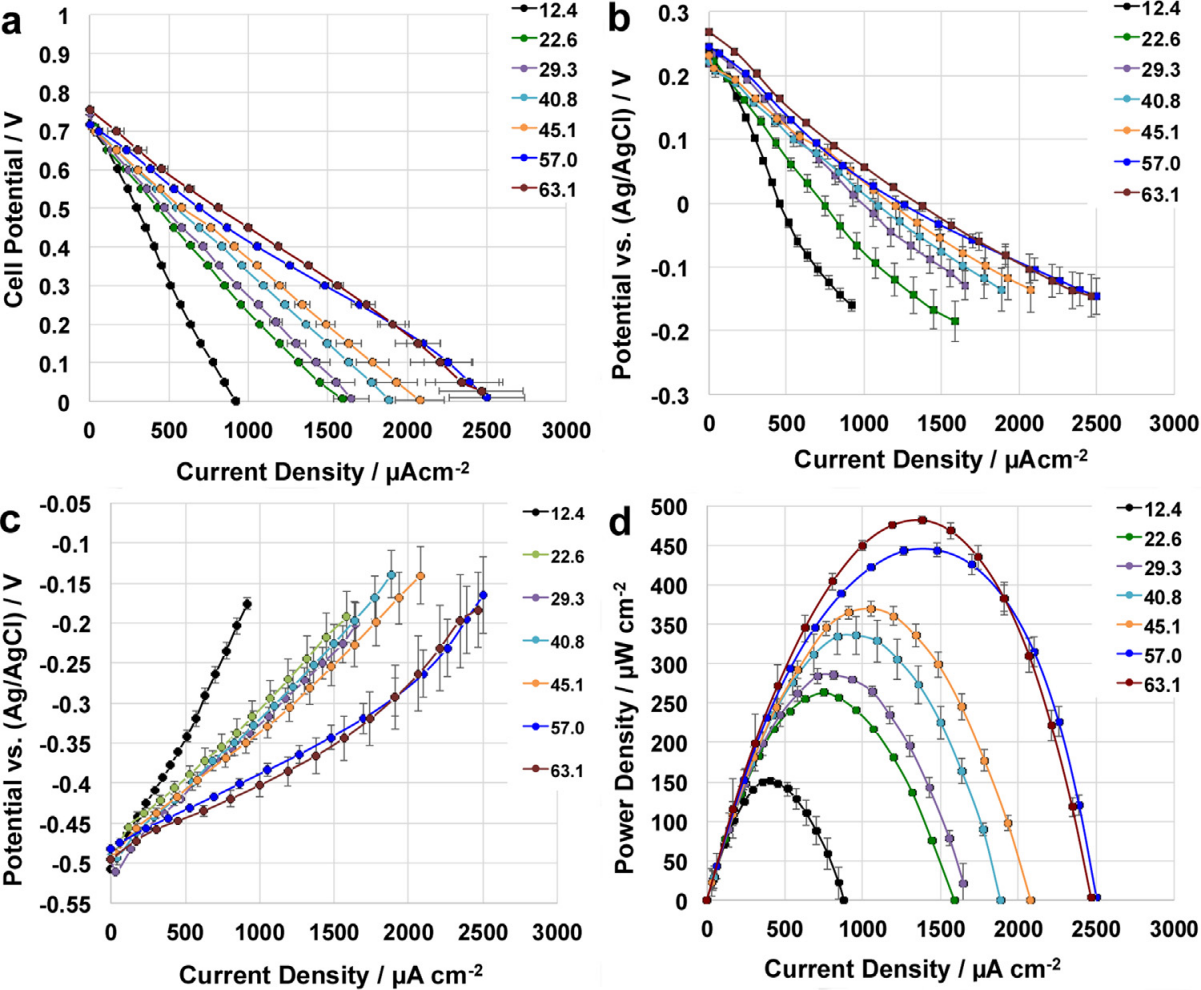
Overall polarization curves (a), cathode (b) and anode (c) polarization curves and power curves (a) of MFC with Fe-AAPyr as cathode catalyst at solution conductivity varying between 12.38 mS cm^−1^ and 63.1 mS cm^−1^.

**Fig. 9:**
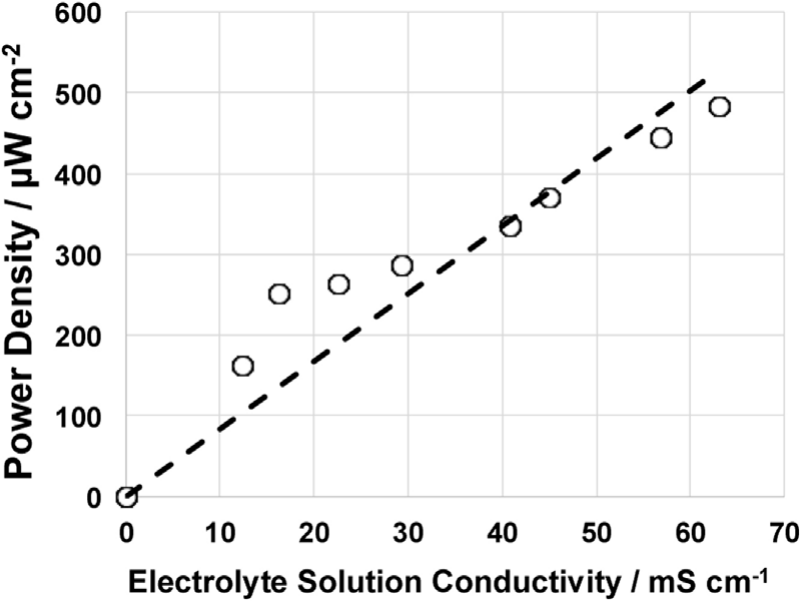
Peak of power density function of the electrolyte solution conductivity.

As it was discussed above, Fe-AAPyr was identified as the most active electro-catalyst among the materials investigated for ORR incorporated into an air-breathing cathode operating in MFC. In this paragraph, the performances of SCMFCs using Fe-AAPyr as cathode catalyst have been investigated varying the solution conductivity of the electrolyte simulating real wastewaters. Particularly, the solution conductivity varied between 12.38 mS cm^−1^ and 63.1 mS cm^−1^. Results showed that the performances increased significantly with the increase in solution conductivity (Fig. 8). Polarization curves showed practically the same open circuit voltage with starting point of 0.742 ± 0.021 V (Fig. 8.a). Different slopes in the polarization curves were identified with the increase in solution conductivity (Fig. 8.a). Separate electrodes polarization curves showed that both anode and cathode polarization curves are positively affected by the electrolyte conductivity (Fig. 8.b and 8.c). In fact, both anode and cathode polarization curves changed their linear slopes positively with the increase in solution conductivity indicating a decrease in ohmic losses with electrolyte conductivity (Fig. 8.b and 8.c). Interestingly, both anode and cathode do not reach diffusion limitation indicating the ohmic losses due to the electrolyte as the main cause of the overall losses. Power density output was then affected positively by the increase in solution conductivity (Fig. 8.d). Results about the power densities obtained are summarized in Table 1. The maximum power recorded was 482 ± 5 μW cm^−2^ for solution conductivity of 63.1 mS cm^−1^ (Fig. 8.d). A quasi-linear increase of the power peak with the solution conductivity in the range investigated can be noticed (Fig. 9). These results underline the important aspect that the performances of the MFCs are electrolyte limited. In fact, the increase in solution conductivity of the electrolyte led to an increase in output.

## 4 Conclusion

For the first time, this study showed a comparison among several ORR PGM-free catalysts having a different metal center and synthesized using consistent preparation method, identical initial ingredients and organic precursors, same cathode structure and tested in repeatable and equal operating conditions. Fe-AAPyr catalysts showed the highest performance compared to Co-AAPyr, Ni-AAPyr, and Mn-AAPyr. The results also indicated a straightforward hierarchy among the PGM-free metal based catalysts in which the metals used during the synthesis affected the performances and obeyed to a certain order: Fe > Co > Ni > Mn. Therefore, Fe should be the metal to utilize during the synthesis of PGM-free catalysts for MFC application. These results indicated clearly also that the metal-based PGM-free catalysts investigated outperformed AC-based (metal-free) cathode by 31-102%. Durability tests results indicated a relatively good stability of Metal-AAPyr with a losses in 32 days quantified in 21-30%. The addition of nonprecious metals catalysts undoubtedly increased the capital cost of the cathode, but this small cost rise (7 cents cm^−2^ [65]) would certainly benefit in enhancing the very low power generated.

A fair and scientifically correct comparison of our current data with existing literature cannot be done since working temperature, configuration utilized, utilization of different electrolytes and different atmospheric conditions (Albuquerque, New Mexico, is at 1500 AMSL with atmospheric pressure reduced by roughly 20% compared to the one on sea level) are quite different and can affect significantly the performances. In other cases, the power was referred to the anode area and not the cathode area with high power output reported [66].

We showed that the increase of the electrolyte solution conductivity increased the performance output considerably with a maximum of 482 ± 5 μW cm^−2^ achieved at the highest solution conductivity investigated. Those results indicated that the MFCs output is severely electrolyte solution limited and consequently the utilization of conductivity waste (e.g. urine [27], [28]) is suggested to increase the output. It must be noticed that also at the highest solution conductivity investigated the overall polarization curves and the anode/cathode polarization curves have a linear trend indicating that they are ohmic-dependent and no diffusion limitation occurs in these conditions. Therefore, a further increase in solution conductivity can be investigated with a probable additional increase in power generated till the plateau is reached. Further directions should certainly pursue the utilization of PGM-free catalysts for MFCs with an additional decrease in catalysts loading to lower the cathode costs.

## Figures and Tables

**Table 1:** Mixture utilized as electrolyte and corresponding solution conductivity measured and power density produced.

K-PB	KCl	AS	SC	Power density
[M]	[M]	% in volume	mS cm^−1^	μW cm^−2^
0.05	0.1	50	12.4	151 ± 2
0.1	0.1	50	16.4	251 ± 2
0.2	0.1	50	22.6	262 ± 4
0.3	0.1	50	29.3	286 ± 6
0.5	0.1	50	40.8	336 ± 20
0.6	0.1	50	45.1	370 ± 9
0.8	0.1	50	57	444 ± 8
1	0.1	50	63.1	482 ± 5

**Table 2:** Elemental composition and chemical speciation of catalysts.

	C 1 s %	O 1 s %	N 1 s %	Metal %	C gr	C-N	C_x_O_y_
Fe-AAPyr	84.8	7.2	7.9	0.25	32.7	13.5	51.3
Co-AAPyr	89.5	8.1	2.3	0.10	50.6	6.4	40.5
Ni-AAPyr	86.3	11.6	2.0	0.19	29.7	17.7	43.3
Mn-AAPyr	82.3	12.5	5.1	0.13	25.0	16.1	51.6

	N pyridinic	N_x_-M	N pyrrolic	N gr	Metal	M-N_x_	MO_x_
Fe-AAPyr	35.9	19.6	25.3	9.5	0.0	28.0	72.0
Co-AAPyr	27.3	12.9	30.3	14.7	11.4	58.4	30.2
Ni-AAPyr	34.7	13.2	28.3	9.7	0.0	16.8	83.2
Mn-AAPyr	35.8	31.5	17.1	6.4	17.8	34.4	47.8

**Table 3:** Onset (E_on_) and half wave potentials (E_1/2_) of the catalysts towards oxygen reduction reaction in neutral media.

	E_on_	E_1/2_
Catalyst	(V vs Ag/AgCl))	(V vs Ag/AgCl))
Fe-AApyr	0.31	0.16
Co-AApyr	0.27	0
Mn-AApyr	0.21	−0.1
Ni-AApyr	0.11	−0.12
AC	0.06	−0.18
