# Supplementary material for: Air Breathing Cathodes for Microbial Fuel Cell using Mn-, Fe-, Co- and Ni-containing Platinum Group Metal-free Catalysts
Source: Electrochim Acta. 2017 Mar 20;231:115–24. doi: 10.1016/j.electacta.2017.02.033 (PMC5384433; doi:10.1016/j.electacta.2017.02.033)
Supplement: Supplementary file 1 [file mmc1.docx]

**SUPPORTING INFORMATION**

**Air Breathing Cathodes for Microbial Fuel Cell using Mn-, Fe-, Co- and Ni-containing Platinum Group Metal-free Catalysts**

Mounika Kodali^1^, Carlo Santoro^1^, Alexey Serov^1^, Sadia Kabir^1^, Kateryna Artyushkova^1^, Ivana Matanovic^1,2^, *Plamen Atanassov^1^

^1^ Center Micro-Engineered Materials (CMEM), Department of Chemical and Biological Engineering, University of New Mexico, Albuquerque, NM, USA

^2^ Theoretical Division, Los Alamos National Laboratory, Los Alamos, NM 87545, USA


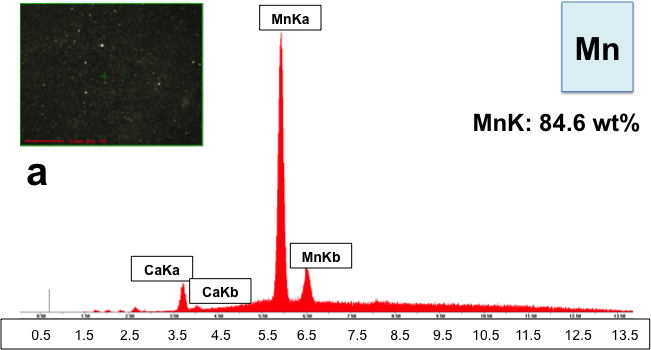


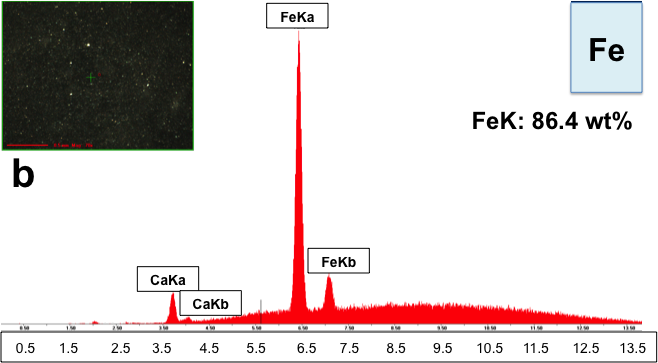


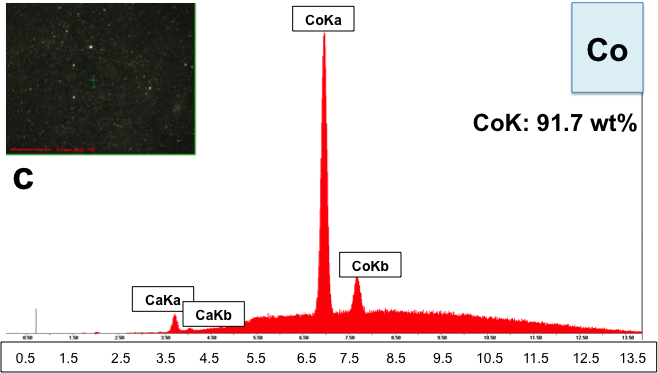


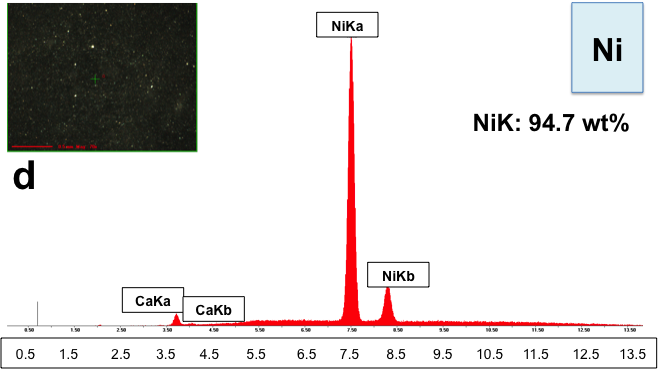


**Figure S1.** Screenshot of the results obtained by XRF

**Figure S2.** High-resolution metal spectra for 4 catalysts.

 **Figure S3.** Density functional theory optimized geometries of Fe-N_4_, Co-N_4_, Ni-N_4_, and Mn-N_4_ centers used to calculate N 1s binding energies. One unit cell is shown.

**Figure S4.** Voltage trend over 32 days of duplicates MFCs having cathodes based on: Fe-AAPyr (a), Co-AAPyr (b), Ni-AAPyr (c), Mn-AAPyr (d) and AC (e).

Few days concerning AC-1 were lost during the recording.

**Figure S5.** Overall polarization curves (a), power curves (b) and anode and cathode polarization curves of MFC with Fe-AAPyr (red), Co-AAPyr (green), Ni-AAPyr (violet), Mn-AAPyr (light blue) and AC (black) as cathode catalyst after 32 days of experiments.


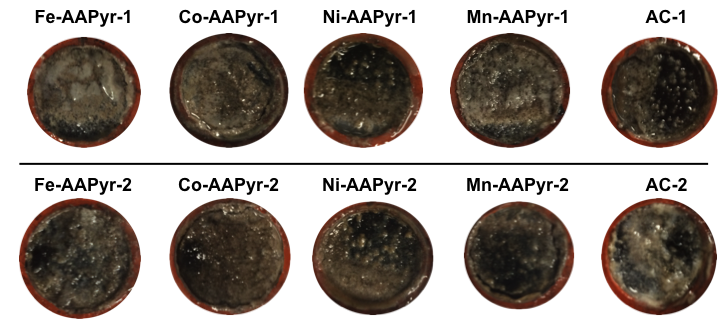


**Figure S6.** Biofilm/fouling formation over the 10 cathodes after 32 days operations
